# Development of the Patient Satisfaction with Medication for Diabetes (PSMD) questionnaire

**DOI:** 10.1186/s41687-026-01037-w

**Published:** 2026-03-25

**Authors:** Kristina S. Boye, Katie D. Stewart, Louis S. Matza, Danielle Soucier-Ernst, Christy Houle, Iris Goetz, Hiren Patel, Chisom Kanu

**Affiliations:** 1 Eli Lilly and Company, Indianapolis, IN, USA; 2 PPD Evidera Patient-Centered Research, Thermo Fisher Scientific, Waltham, MA, USA

**Keywords:** Type 1 diabetes, Type 2 diabetes, Treatment satisfaction, Patient Satisfaction with Medication for Diabetes, PSMD

## Abstract

**Background:**

Recent advances in treatment for type 1 (T1D) and type 2 diabetes (T2D) may have an impact on patients’ treatment satisfaction, which can affect adherence and outcomes. Established patient-reported outcome (PRO) measures of treatment satisfaction may not be sensitive to the treatment attributes and outcomes that are relevant and important to patients receiving new diabetes medications. The purpose of this study was to develop a new PRO measure to assess treatment satisfaction that would be relevant to patients receiving various diabetes treatments, including newer medications.

**Methodology:**

Concept elicitation interviews were conducted with adults with T1D and T2D to identify factors that contribute to treatment satisfaction. Results of these interviews were used to generate content for a draft PRO measure. Cognitive interviews were conducted with patients with T1D and T2D to evaluate the draft questionnaire.

**Results:**

Concept elicitation interviews were conducted with participants with T1D (*n* = 10; mean age = 41.7 years; 70% female) and T2D (*n* = 20; mean age = 61.2 years; 45% female). Patients reported treatment attributes that contribute to satisfaction, such as medication effectiveness, medication administration, and impact on energy, weight, and eating behavior. The new PRO measure, the Patient Satisfaction with Medication for Diabetes (PSMD™) questionnaire, was evaluated in cognitive interviews with participants with T1D (*n* = 5; mean age = 40.8 years; 60% female) and T2D (*n* = 10; mean age = 60.8 years; 60% female). Results of the cognitive interviews suggest that the final instrument is clear, comprehensible, and relevant to patients.

**Conclusions:**

Results support the content validity of the PSMD, which may be useful in assessing patients’ satisfaction with new and established diabetes medications.

## Introduction

Treatments for type 1 (T1D) and type 2 diabetes (T2D) vary with regard to outcomes like glycemic control and weight loss, as well as in aspects of the treatment process such as mode of administration, dose frequency, and injection devices. Differences in medication regimens and clinical outcomes can influence a patient’s satisfaction with their diabetes treatment [1–5]. Greater treatment satisfaction can lead to better treatment adherence [6–10], which has a positive impact on health outcomes [11–13].

Satisfaction with diabetes treatment can be assessed using a range of generic measures, such as the Treatment Satisfaction with Medicines Questionnaire (SATMED-Q) [14, 15] and the Treatment Satisfaction Questionnaire for Medication (TSQM) [16–21]. However, these generic instruments that were designed to apply to patients across medical conditions do not focus on the specific experiences of patients with diabetes. Therefore, condition-specific measures are often used to assess treatment satisfaction in this population.

Several diabetes-specific patient-reported outcome (PRO) measures have been used to assess overall treatment satisfaction, including the Diabetes Treatment Satisfaction Questionnaire [22, 23], Diabetes Medication Satisfaction [24], Diabetes Quality of Life Clinical Trial Questionnaire [25], Diabetes Quality of Life [26], Insulin Delivery System Rating Questionnaire [27], and Inhaled Insulin Treatment Questionnaire [28]. In addition, some measures have subscales or items focusing on satisfaction with specific treatment attributes, such as clinical effects [24, 29–31], emotional impact [24, 32], and aspects of the treatment process [24, 29, 30, 33, 34].

The landscape of diabetes treatment has changed significantly in recent years with the introduction of new medications like glucagon-like peptide-1 (GLP-1) receptor agonists and new treatment administration approaches. Established PRO measures may not assess satisfaction with specific outcomes and treatment administration attributes that are relevant to patients receiving these new medications. For example, existing measures often omit key features that differentiate newer treatments, such as weight loss, ease of administration, and dose frequency [23, 24, 26–29]. Furthermore, several measures include items that are only relevant to specific types of medication [27, 28, 32, 35, 36] or routes of administration [33, 34], which limits their applicability across treatments.

To be useful in current clinical trials, a diabetes treatment satisfaction questionnaire should assess concepts relevant to the newer treatment options and exclude items that would only be relevant to previous treatments. Therefore, the purpose of the current study was to address these measurement gaps by developing a new PRO instrument to assess treatment satisfaction for patients with diabetes. This measure, called the Patient Satisfaction with Medication for Diabetes (PSMD) questionnaire, was designed to include key concepts identified as important by a range of patients with diabetes, including patients receiving newer injectable treatments. The current study focused on the initial qualitative phases of instrument development, including concept elicitation and cognitive interviews with adults with T1D and T2D. This study was designed in accordance with the Food and Drug Administration’s (FDA) recommendations in the 2009 PRO Guidance for Industry [37] and the Patient-Focused Drug Development Guidance [38].

## Methods

### Overview of study steps

**Fig. 1:**
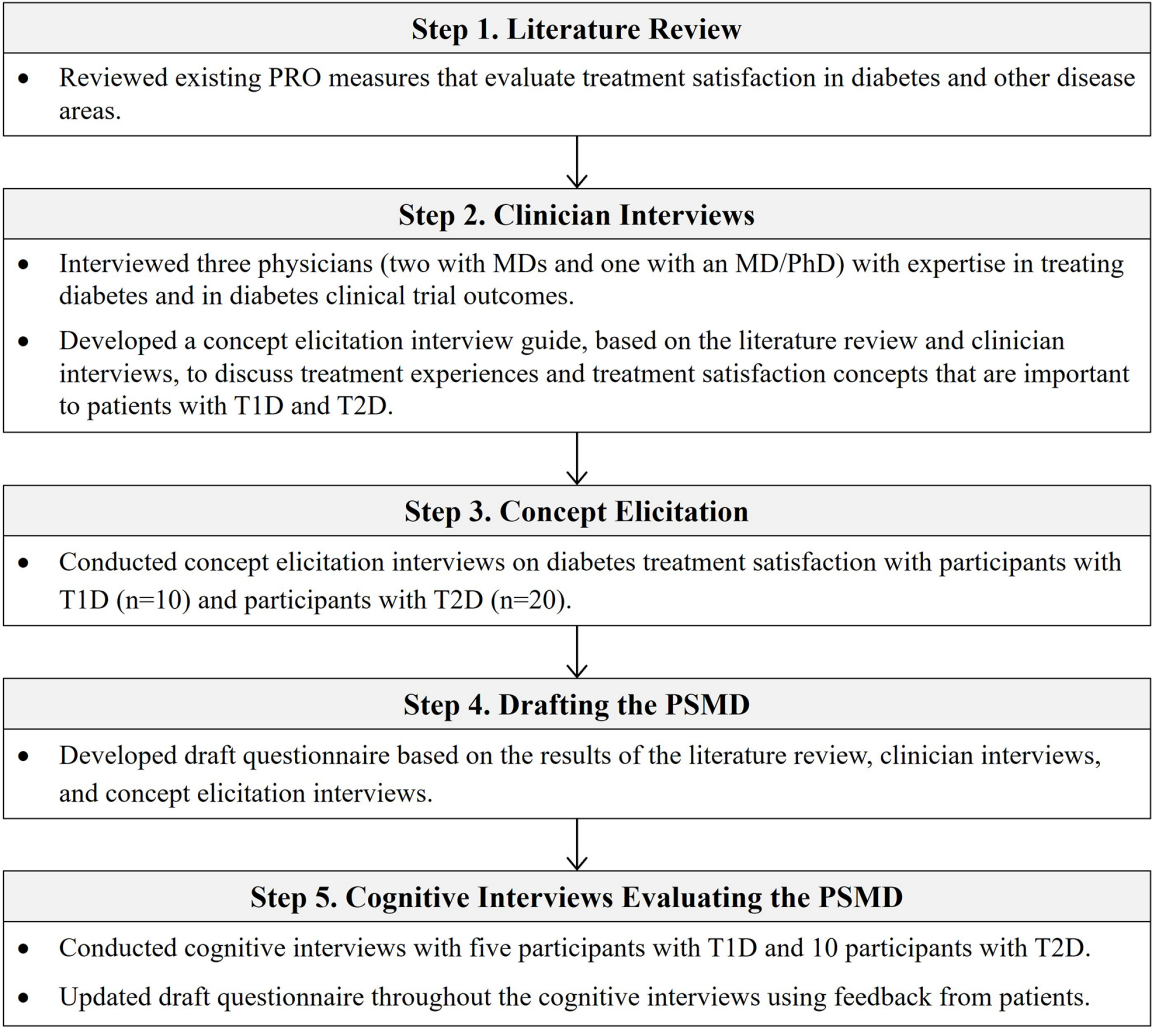
Summary of instrument development of the PSMD. Abbreviations: PRO = patient-reported outcome; PSMD = Patient Satisfaction with Medication for Diabetes; T1D = type 1 diabetes; T2D = type 2 diabetes

The PSMD questionnaire was developed in a series of five steps summarized in Fig. 1. First, previously published PRO measures assessing treatment satisfaction in diabetes and other disease areas were reviewed to identify relevant concepts for inclusion in the concept elicitation interview guide. In step 2, interviews were conducted with three clinicians (two MDs and one MD/PhD) with expertise in diabetes treatment and research. All three endocrinologists had over 10 years of experience treating T1D and T2D and regularly saw patients despite their research focus. These interviews focused on identifying concepts related to patient satisfaction with diabetes treatment. The concepts identified in the review of PRO measures (step 1) and clinician interviews (step 2) informed the development of the concept elicitation interview guide for the next step.

In step 3, concept elicitation interviews were conducted with patients with T1D and T2D to identify concepts that contribute to treatment satisfaction among patients with diabetes. In step 4, the results of these qualitative interviews were considered along with the previous steps when developing a draft version of the new PRO measure. In step 5, participants with T1D and T2D completed the draft questionnaire and provided feedback on its clarity, comprehensibility, comprehensiveness, and relevance in cognitive interviews.

Throughout step 5, feedback from participants was used to refine the draft questionnaire. All study methods, materials, and clinical sites were approved by an independent review board (Salus IRB, study number 24116). All participants provided informed consent before engaging in study procedures. The consent form notified participants that their data may be published, but they would not be personally identified in any publications. All participants were compensated for their time.

### Participants

Steps 3 and 5 included interviews with people living with T1D or T2D. These participants were required to be (1) ≥ 18 years of age; (2) diagnosed with T1D or T2D by a recognized medical professional at least 6 months before the interview; (3) treated with medication for diabetes for at least 3 months before the interview; (4) residing in the United States; (5) willing and able to provide consent for participation; and (6) willing and able to complete protocol requirements. Potential participants were excluded if they (1) had a diagnosis of latent autoimmune diabetes or gestational diabetes; (2) had cognitive impairment, hearing difficulty, visual impairment, severe psychopathology, or insufficient knowledge of the English language that could interfere with their ability to provide consent and complete the study interview and measures; or (3) were employed by a pharmaceutical company or had a direct role in treating patients with diabetes.

Participants were recruited by US-based clinical sites. Potential participants were identified from each clinic’s medical records, and the clinic staff approached potentially eligible patients during a clinical visit or called via telephone to introduce the study and assess interest in participation. Participants in the concept elicitation interviews (step 3) were recruited from four clinical sites (Miami, FL; Humble, TX; Fargo, ND; Rochester, NY), and participants in the cognitive interviews (step 5) were recruited from five clinical sites (Miami, FL; Humble, TX; Fargo, ND; Rochester, NY; Smithfield, PA).

In qualitative research, the sample size is considered adequate when data “saturation” has been reached. Saturation is defined as the point at which no substantially new concepts of interest (i.e., symptoms or impacts) emerge as additional interviews are conducted [39]. The number of participants needed to reach saturation is largely driven by the complexity of the concepts being studied and the diversity of the participant population sharing the condition, treatment, or other health-related experience of interest. A review of 26 concept elicitation studies found that approximately 85% of concepts were typically identified after 10 interviews, more than 90% after 15 interviews, and more than 95% after 20 interviews [40]. A target sample size of 30 participants was selected for the current concept elicitation study. For the cognitive interviews, a target sample size of 15 participants is within the range recommended by best practice guidance for this type of qualitative study [41–43].

### Interview procedures

#### Concept elicitation interviews (Step 3)

The concept elicitation interviews were conducted from April to July 2024 by four interviewers trained in the study procedures and qualitative research methods. Participants had the option to be interviewed via telephone or videoconference, and all participants chose to complete the interview by telephone. Interviews followed a semi-structured interview guide to elicit discussion of patients’ experiences with diabetes treatment and factors contributing to treatment satisfaction. The interview began with general questions about satisfaction with treatment for diabetes without suggesting specific aspects of treatment that could have contributed to satisfaction (e.g.,* Are you satisfied with your current treatment for diabetes? Why or why not? What aspects of treatment are you satisfied/dissatisfied with? Were you satisfied with your previous treatment? What led you to change treatment?*). The interview continued with questions on satisfaction with specific aspects of treatment, including effectiveness, administration, impact on daily activities, weight, and other aspects of treatment that respondents reported as contributing to their satisfaction or dissatisfaction.

#### Cognitive interviews (Step 5)

The cognitive interviews were conducted by three trained interviewers between November and December 2024 according to a semi-structured interview guide. All interviews were conducted via a web-based conference platform that allowed the interviewer to share their screen (i.e., Microsoft Teams). Participants completed the draft PSMD and were interviewed about their understanding and interpretation of the instructions (*What are the instructions asking you to do?*), recall period (*What does the timeframe “in the past 7 days” mean to you?)*, response options (*Were these response options clear to you?*), item stem (*What does this mean to you? Is it clear to you that this phrase is the first part of each question?*), and items (*In your own words*,* what does this question mean to you? What is it asking?*). Participants were asked to explain their reasons for selecting each response (*What answer did you choose and why?*) and if it was easy to respond to each item (*Was it easy to respond to this item? If not easy*,* why?*). Participants were then asked about the relevance of the items (*Were there any questions that should not be asked?*) and the comprehensiveness of the questionnaire (*Are there any questions that we should ask that are not on the questionnaire? What other things should we ask about? Were there any questions missing from this questionnaire?*). Participants were also asked about the formatting of the questionnaire *(Is the format [how the questions and answer choices look on the page] easy or difficult to follow? Is the ordering of the items easy to follow?*).

### Measures

All participants completed a brief sociodemographic questionnaire including items on age, gender, living situation, employment, education level, racial/ethnic background, and general health. Site personnel completed a clinical information form for each participant. This form included questions to document the participant’s diabetes diagnosis date, current medications for diabetes, most recent glycated hemoglobin (HbA1c) value, and body mass index.

### Analysis procedures

#### Quantitative analysis

Responses to demographic and clinical questions were summarized with descriptive statistics (means and standard deviations for continuous variables; frequencies and percentages for categorical variables).

#### Qualitative analysis

Interviews in steps 3 and 5 were recorded and transcribed for subsequent qualitative analysis. The transcripts were coded using ATLAS.ti (version 22) following a content analysis approach [44]. Coding was completed according to a dictionary of codes and definitions of each code, which were developed based on themes, concepts, and terms from the interview guides. Additional concepts that emerged throughout coding were added to these dictionaries.

Coding was performed by researchers trained in qualitative analysis methods and ATLAS.ti coding. During the coding process, quotations describing participants’ treatment experiences were coded so that related statements could be grouped by concept and theme. These concepts and themes were subsequently used to generate items for the new questionnaire. Two staff members coded the transcripts from the step 3 interviews, and three staff members coded the transcripts from the step 5 interviews. The first transcript from each step was coded by all coders, and the coding was reviewed for discrepancies. The coders then met to discuss and resolve these discrepancies to better align their coding decisions. The coding team continued coding additional transcripts independently, and coding results were compared until the inter-coder agreement exceeded 80%. After acceptable inter-coder agreement was achieved, the remaining transcripts were each coded by one of the coders. A senior staff member performed a quality review of the coding for all transcripts.

Transcripts of the concept elicitation discussions (step 3) were coded by selecting words and phrases based on the coding dictionary and grouping these qualitative data into key concepts. To examine saturation, data were summarized in a grid according to FDA guidance [38].

The analysis of data from the cognitive interviews (step 5) included assessment of the percentage of participants who understood each item, as well as the percentage who had difficulty understanding the instructions, recall period, response options, or item stem. Analyses also focused on identifying concepts missing from the questionnaire that participants said were relevant to their satisfaction with treatment for diabetes.

## Results

### Literature search (Step 1) and clinician interviews (Step 2)

The literature search identified 16 PRO measures assessing treatment satisfaction in diabetes. These measures were reviewed for relevance to new and emerging diabetes medications. However, none of the instruments were found to be suitable for these newer treatments, because of the omission of important concepts [23, 24, 26–29], inclusion of items that are only relevant to specific types of treatment [27, 28, 32–36], lack of specificity in response scales [25, 33, 35], focus on treatment burden rather than treatment satisfaction [24, 31], and lack of evaluation of overall treatment satisfaction [30, 45].

Ten measures used to assess treatment satisfaction in other disease areas were also reviewed for relevant concepts, including measures designed for obesity [46, 47], sleep apnea [48, 49], osteoarthritis [50–53], erectile dysfunction [54], and pulmonary fibrosis [55]. In addition, two commonly used generic measures of treatment satisfaction were examined (the TSQM [16] and SATMED-Q [15]). These questionnaires included several concepts in common with the diabetes treatment satisfaction PROs, such as ease of use [51, 54, 55], treatment convenience [51], side effects [51, 55], and emotional impact [55].

In the clinician interviews, a range of concepts related to treatment satisfaction emerged. These concepts can be grouped into three broad categories: treatment effectiveness, treatment administration attributes, and impact on daily life. The clinicians also shared insight into how treatment satisfaction may be similar or different for patients with T1D and T2D. The concepts identified in the literature search and clinician interviews informed the development of the qualitative interview guide used in step 3 and the draft measure in step 4.

### Concept elicitation interviews (Step 3)

Concept elicitation interviews were conducted with 10 patients with T1D and 20 patients with T2D (sample characteristics in Table 1). Most participants (T1D 80%; T2D: 90%) were satisfied with their diabetes treatment. Participants were asked which aspects of treatment generally contribute to satisfaction or dissatisfaction with medication, and the most commonly reported aspects are presented in Table 2.

**Table 1:** Participant characteristics

Characteristics	Concept Elicitation Interviews (Step 3)			Cognitive Interviews (Step 5)	
	T1D (*N* = 10)	T2D (*N* = 20)		T1D (*N* = 5)	T2D (*N* = 10)
**Age in years**,** mean (SD) [range]**	41.7 (18.4) [21.0–74.0]	61.2 (9.4) [45.0–78.0]		40.8 (23.6) [20.0–67.0]	60.8 (8.9) [46.0–74.0]
**Gender**,** n (%)**					
Male	3 (30.0%)	11 (55.0%)		2 (40.0%)	4 (40.0%)
Female	7 (70.0%)	9 (45.0%)		3 (60.0%)	6 (60.0%)
**Ethnicity**, **n (%)**					
Hispanic or Latino	3 (30.0%)	5 (25.0%)		2 (40.0%)	-
Not Hispanic or Latino	7 (70.0%)	14 (70.0%)		3 (60.0%)	10 (100.0%)
Missing	-	1 (5.0%)		-	-
**Race**, **n (%)**					
American Indian or Alaska Native	-	-		-	1 (10.0%)
Black or African American	2 (20.0%)	6 (30.0%)		1 (20.0%)	3 (30.0%)
Native Hawaiian or other Pacific Islander	-	1 (5.0%)		-	-
White	7 (70.0%)	11 (55.0%)		4 (80.0%)	5 (50.0%)
Multiple races^a^	1 (10.0%)	1 (5.0%)		-	-
Other	-	1 (5.0%)		-	1 (10.0%)
**Employment status**,** n (%)**					
Full-time work	6 (60.0%)	8 (40.0%)		2 (40.0%)	3 (30.0%)
Part-time work	2 (20.0%)	3 (15.0%)		1 (20.0%)	2 (20.0%)
Other^b^	2 (20.0%)	9 (45.0%)		2 (40.0%)	5 (50.0%)
**Education level**,** n (%)**					
University degree	5 (50.0%)	3 (15.0%)		1 (20.0%)	3 (30.0%)
No university degree	5 (50.0%)	16 (80.0%)		4 (80.0%)	7 (70.0%)
Missing	-	1 (5.0%)		-	-
**Marital status**,** n (%)**					
Single	4 (40.0%)	4 (20.0%)		2 (40.0%)	-
Married/Cohabitating/Living with partner	5 (50.0%)	12 (60.0%)		1 (20.0%)	7 (70.0%)
Other^c^	1 (10.0%)	3 (15.0%)		2 (40.0%)	3 (30.0%)
**Duration of diabetes diagnosis in years**,** mean (SD)**	23.2 (18.3) [2.0-50.3]	13.9 (10.0) [1.5–32.9]		26.7 (23.1) [2.9–62.4]	13.7 (12.3) [0.7–34.4]
**Current treatment for diabetes**,** n (%)**					
Oral medication only	-	10 (50.0%)		-	4 (40.0%)
Injectable insulin only	3 (30.0%)	-		2 (40.0%)	1 (10.0%)
Insulin pump only	-	-		1 (20.0%)	-
Oral medication and injectable insulin	-	1 (5.0%)		-	-
Oral medication and injectable GLP-1 RA	-	2 (10.0%)		-	3 (30.0%)
Oral medication and injectable GIP/GLP-1 RA	-	3 (15.0%)		-	1 (10.0%)
Oral medication, injectable insulin, and injectable GLP-1 RA	-	1 (5.0%)		-	-
Oral medication, injectable insulin, and injectable GIP/GLP-1 RA	-	2 (10.0%)		-	1 (10.0%)
Oral medication, injectable insulin, injectable GIP/GLP-1 RA, and insulin pump	-	1 (5.0%)		-	-
Injectable insulin and insulin pump	6 (60.0%)	-		2 (40.0%)	-
Injectable insulin, injectable GLP-1 RA, and insulin pump	1 (10.0%)	-		-	-
**HbA1c (%)**,** mean (SD) [range]**^d^	8.9 (1.7) [5.6–11.2]	7.6 (1.8) [5.5–13.0]		7.6 (2.1) [5.3–10.2]	6.6 (1.2) [4.9–9.3]
**BMI (kg/m**^**2**^**)**,** mean (SD) [range]**	27.8 (4.6) [20.2–36.2]	35.6 (6.7) [27.0-55.5]		30.3 (7.3) [24.0-42.1]	31.2 (4.4) [26.6–37.9]

Abbreviations: BMI = body mass index; GIP = glucose-dependent insulinotropic polypeptide; GLP-1 = glucagon-like peptide-1; HbA1c = glycated hemoglobin; RA = receptor agonist; SD = standard deviation; T1D = type 1 diabetes; T2D = type 2 diabetes

^a^Multiple races (concept elicitation): One participant with T1D and one participant with T2D indicated both American Indian or Alaska Native and White

^b^Other employment statuses (concept elicitation) include homemaker (*n* = 1 T2D), student (*n* = 1 T1D), unemployed (*n* = 1 T1D), disabled (*n* = 2 T2D), and retired (*n* = 6 T2D). Other employment statuses (cognitive interviews) include student (*n* = 1 T1D), unemployed (*n* = 1 T2D), disabled (*n* = 1 T2D), and retired (*n* = 1 T1D; *n* = 3 T2D)

^c^Other marital statuses (concept elicitation) include divorced (*n* = 1 T1D). Other marital statuses (cognitive interviews) include divorced (*n* = 1 T1D; *n* = 2 T2D) and widowed (*n* = 1 T1D; *n* = 1 T2D)

^d^Results of the most recent HbA1c test were unknown for one concept elicitation interview participant (T1D) and one cognitive interview participant (T1D)

**Table 2:** Treatment satisfaction concepts frequently reported during concept elicitation interviews in step 3

Aspects of Treatment Contributing to Satisfaction or Dissatisfaction	T1D (*N* = 10) *n* (%)^a^	T2D (*N* = 20) *n* (%) ^a^	Example Quotations
Effectiveness of medication	6 (60%)	20 (100%)	“Effectiveness, how fast it works, and how well and efficient it works… I want to stay pretty stable. I don’t want it to go up and then crash down. If the medication isn’t really effective, I don’t see it as a satisfying medication.” (M, 22y, T1D)
Medication administration	10 (100%)	12 (60%)	**Mode of administration**: “I’m used to giving myself shots, so that’s super, super easy. Now, the oral medication that I’m on, it is kind of a big pill, so it’s kind of nasty to swallow because it is kind of big. I wish there was maybe something smaller… If she could just give me that one in an injectable too, I would really appreciate it more than having to take it orally.” (F, 56y, T2D)^b^ **Ease of medication preparation**: “It’s basically no preparation other than cleansing this area with an alcohol wipe.” (F, 65y, T2D) **Simplicity/complexity/ease of use**: “Just how easy it is to use. It’s not complicated.” (M, 22y, T1D) **Frequency of medication administration**: “It was the constant injections… Three to four times a day. Sometimes, more. It made things uncomfortable.” (M, 29y, T1D) **Forgetting/remembering to take medication**: “I’m pretty satisfied…A lot of times, I’ve forgotten my insulin pens at home; it’s absurd. And having them on me, like in the form of a pump, has made my life a bajillion times easier.” (F, 21y, T1D) **Privacy when administering medication**: “I have to go to a different area to inject myself. I have to raise up my shirt to inject myself in the stomach. It’s complex… Especially at work. Since my uniform has to be tucked in at all times. So I have to go to the bathroom and tuck it back in and go out.” (M, 22y, T1D) **Dose adjustments**: “I take action with my pump every single time I put something in my mouth with any kind of carb content. So that usually happens multiple times a day…I wish I had something that would just read my blood sugar levels and give me the insulin I need. … The fact that I have to dial up an amount of insulin every time I put something in my mouth is not exactly ideal.” (F, 60y, T1D)^b^ **Convenience**: “The injectables were fine because I was able to just, on my own time, to do the injectables here at home. Now, one of the oral medications I was on, I had to take it in the morning without food and just a little bit of water and stuff. And that was kind of hard for me because I have to go to work and I couldn’t eat. After I took the medication, I couldn’t eat for like after an hour. That was one of the things I didn’t like.” (F, 56y, T2D)^b^ **Supplies**: “The only issue that I have is getting my pump supplies on time, and that has created some problems…. Things don’t ever come when they’re supposed to. And that’s left me in some pretty precarious situations where I haven’t had the supplies I needed to make my pump run the way it’s supposed to.” (F, 60y, T1D)^b^
Side effects	1 (10%)	8 (40%)	“I’m satisfied. I didn’t have any negative results from taking the oral. I never had a negative result, like upset stomach or vomiting, as those were some of the side effects of it.” (F, 64y, T2D)
Energy	1 (10%)	5 (25%)	“With this new medication, I’m feeling a 100% better. I don’t feel tired. With the medication I was taking before, I guess because the sugar level was so high, I was coming home tired. I was feeling bad at work. But with this one, I’m fine. I’m coming home and I got the energy to do things around my house. So I’m very satisfied.” (M, 63y, T2D)
Weight	2 (20%)	8 (40%)	“With the weight loss, I have gotten so much more mobile and I’ve been able to function in my daily life… So that had a huge impact with my satisfaction because I was able to lose that weight and was able to do things. So I was able to go on a cruise and walk. So that was just in my path life-changing.” (F, 65y, T2D) “The medication that I take that made it so easy for me to lose weight, that was huge for me… But for me, in particular, was always something that I struggled with. So being able to maintain a current healthy weight is a big thing, and something that I’m satisfied with, with the current medication that I take.” (F, 60y, T1D)^b^ “I was not satisfied…I gained weight, even if I was I was even trying to eat properly. The weight was not coming off.” (F, 64y, T2D)
Health (other than blood glucose)	2 (20%)	10 (50%)	“Just I feel better. I feel like sleeping better. I feel like more awake.” (M, 45y, T2D)^b^ “My health, I feel healthy with that medicine. I say wow, that medicine helped me a lot, so it’s good for me because I feel healthy… It impacted in a lot of areas in my body. My legs, before when I got treatment, they hurt, and my arms and everything hurt before, and now I feel strong, and I do exercise, I do everything, and I feel good.” (F, 59y, T2D)
Impact	3 (30%)	3 (15%)	**Daily activities**: “Started out like every month, and I’m out three days. I’m in bed, I can’t go anywhere or do anything. I can’t do stuff around my house… I can’t do household chores and clean and cook or anything.” (F, 55y, T2D) **Daily activities**: “It’s impacted in a very good way. I can do my work without worrying about, as I said, eating. I’m satisfied. I’m very satisfied, that I can move on doing other stuff, going on my errands and not worrying about a specific time I have to eat. It just keeps me going.” (F, 64y, T2D) **Social activities or relationships**: “I’m going to say it makes you more sociable. It does increase your energy when your diabetes ain’t too high because when it’s too high, you get sluggish.” (M, 67y, T2D) **Social activities or relationships**: “I don’t have to worry about stopping to eat or to drink. Or I can have a conversation. I am happy. So it makes everything in me happy. I’m in a happy mood. So my relationship, it’s never been bad, but it’s better.” (F, 64y, T2D)
Appetite and eating behavior	3 (30%)	6 (30%)	“With the injectable [current medication], I did take off a lot of weight. And as I said, the hunger management is great. I’m very satisfied with the results…. It [previous medication] still wasn’t doing what it’s supposed to…I was not satisfied with it because I was still hungry and my sugar levels were still going up.” (F, 64y, T2D)

Abbreviations: F = female; M = male; T1D = type 1 diabetes; T2D = type 2 diabetes; y = years

^a^This column presents the number and percentage of participants who reported satisfaction or dissatisfaction with the specified aspect of their diabetes treatment

^b^The sample included four sets of participants with the same combinations of age, gender, and diabetes type, including M, 45y, T2D (*n* = 2); F, 56y, T2D (*n* = 2); F, 60y, T1D (*n* = 2); and M, 72y, T2D (*n* = 2)

In addition, participants were asked what makes them satisfied or dissatisfied with a range of specific treatment attributes suggested by the interviewer. For example, when asked what makes them satisfied or dissatisfied with treatment effectiveness, participants discussed satisfaction associated with blood glucose control (mentioned by 100% of participants with T1D and 95% with T2D), HbA1c (70% T1D; 65% T2D), how long it takes for the medication to work (30% T1D; 10% T2D), and general effectiveness (e.g., “it works well”) (70% T1D; 90% T2D). When asked about medication administration, participants most commonly discussed satisfaction with mode of administration (mentioned by 80% of participants with T1D and 95% with T2D), simplicity/complexity/ease of use (100% T1D; 95% T2D), ease of medication preparation (90% T1D; 85% T2D), frequency of administration (100% T1D; 100% T2D), and convenience (80% T1D; 30% T2D). Most administration issues were important to participants with both T1D and T2D, but three issues were mentioned more frequently by participants with T1D: automation (80% T1D; 25% T2D), dose adjustments related to food (20% T1D; 0% T2D), and supplies (30% T1D; 5% T2D).

All participants described at least one way that they were satisfied or dissatisfied with the impact of diabetes treatment on their lives. For example, they described the impact on daily activities (mentioned by 100% of participants with T1D and 95% with T2D) and the impact on relationships and social activities (40% T1D; 90% T2D). Many participants reported satisfaction or dissatisfaction with treatment attributes that are often associated with newer medication classes, such as GLP-1 receptor agonists [56–60], including impact on weight (100% T1D; 90% T2D), appetite/eating behavior (100% T1D; 100% T2D), and energy (20% T1D; 35% T2D).

All concepts related to treatment satisfaction endorsed by more than one participant arose within the first 13 interviews, and no new concepts emerged in the final three interviews. Therefore, it was determined that saturation had been reached, and no additional concept elicitation interviews were needed.

### Drafting the questionnaire (Step 4)

**Fig. 2:**
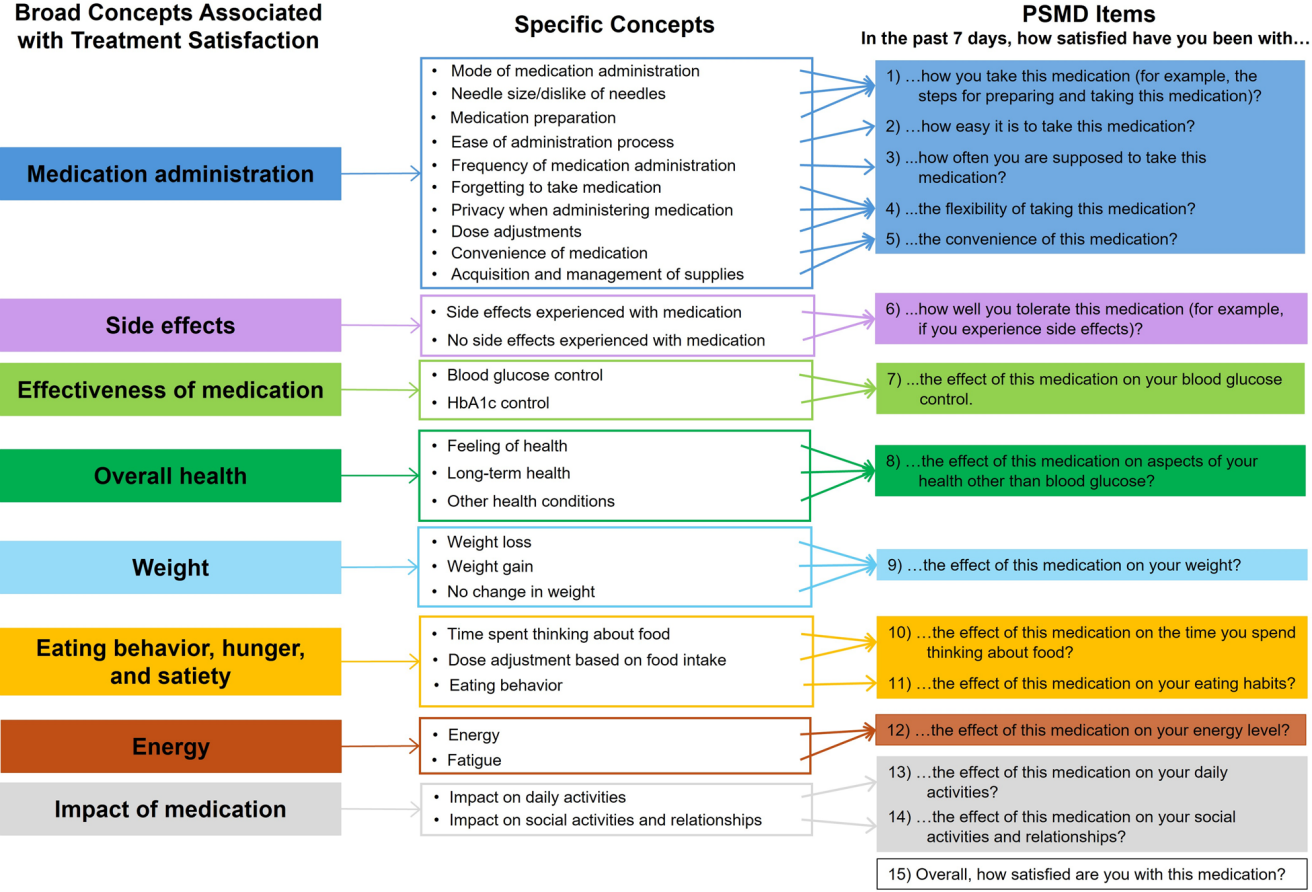
Diabetes treatment satisfaction conceptual framework. Abbreviations: HbA1c = glycated hemoglobin; PSMD = Patient Satisfaction with Medication for Diabetes

The concepts identified in the review of existing treatment satisfaction measures, clinician interviews, and concept elicitation interviews with patients were used to develop a conceptual framework of satisfaction with diabetes treatment (Fig. 2) and the new draft PRO. The PSMD was developed to assess satisfaction with aspects of treatment that are important to patients with diabetes, including patients treated with the newer classes of medications (e.g., GLP-1 receptor agonists).

The study team considered developing separate items for patients with T1D and T2D. However, the concept elicitation results indicated that the treatment attributes considered by patients when evaluating treatment satisfaction did not differ substantially by type of diabetes. Therefore, only one version of the questionnaire was designed, and it is applicable to patients with either T1D or T2D. Patients with T1D and patients with T2D may interpret some concepts differently, depending on their treatment experience and diabetes management. For example, when responding to an item on “time spent thinking about food,” respondents with T1D may consider counting carbohydrates and adjusting their insulin dosage, while respondents with T2D may focus on appetite and food noise (i.e., intrusive, persistent thoughts about food) [61]. The interpretation of each item was assessed when evaluating the content validity of the draft instrument in step 5.

### Cognitive interviews assessing the PSMD (Step 5)

Cognitive interviews were conducted with 15 participants (T1D *n* = 5; T2D *n* = 10) to evaluate the draft PSMD (sample characteristics are presented in Table 1). Participants were receiving a range of treatments for diabetes (Table 1). Participants with T1D were receiving insulin via only injections (40.0%), only an insulin pump (20%), or a combination of injections and pump (40%). Participants with T2D were receiving only oral medication (40%), only injectable insulin (10%), or oral medication combined with the following treatments: an injectable GLP-1 receptor agonist (30%), an injectable dual glucose-dependent insulinotropic polypeptide (GIP)/GLP-1 receptor agonist (10%), or insulin and an injectable GIP/GLP-1 receptor agonist (10%).

Each participant was asked to complete the draft PSMD while focusing on one of their current medications for diabetes. There were four rounds of interviews in total (i.e., two interviews in the first round, six in the second, four in the third, and three in the fourth). Prior to the second, third, and fourth rounds, the PSMD was slightly revised to incorporate feedback from the previous round of interviews. Participants consistently understood the instructions, recall period, item stem, and response options. Across the full cognitive interview sample, all seven response options were used.

Table 3 presents example quotations illustrating participants’ interpretations of the PSMD items. Most items were interpreted as intended by all participants. For example, when responding to the item on satisfaction with blood glucose control, all participants reported thinking about their blood glucose numbers from self-monitoring or HbA1c tests. However, interpretations of some items were more variable, reflecting the broad range of issues that are important to patients with diabetes. For example, when responding to the item on medication flexibility, participants considered flexibility in timing (T1D 60%; T2D 100%) and location (T1D 60%; T2D 10%), as well as flexibility to take the medication later if forgotten (T1D 40%; T2D 80%) or as needed to control blood sugar (T1D 40%; T2D 0%). The item assessing the more general concept of “flexibility” was retained to allow patients to interpret the item in a way that is relevant to their current treatment. When responding to the item on thinking about food, participants reported considering time spent thinking about food and eating in general (T1D 80%; T2D 80%), planning medication administration around food (T1D 60%; T2D 20%), planning food intake around medication (T1D 40%; T2D 10%), and dose calculations related to food (T1D 20%; T2D 0%).

**Table 3:** Example quotations demonstrating participants’ interpretations of PSMD items

Items^a^	Example Quotations of Participants' Interpretations of the Item^b^
1) …how you take this medication (for example, the steps for preparing and taking this medication)?	“Am I satisfied with preparation I have to go through with cleaning my arm, the injection, that sort of thing. Yeah, I am very satisfied.” (F, 59y, T2D)^c^
2) …how easy it is to take this medication?	“It’s asking how easy is it to take the medication?… I chose…extremely satisfied because it is easy. It’s an injection, but it’s easy to take the medication.” (F, 59y, T2D)^c^ “This question is asking me if I had any trouble taking it, if I understood how easy it is to take the dosage. I said extremely satisfied… For me, it’s very easy to take. I mean, it’s a pill. You just take it with your water in the morning and water at night.” (F, 64y, T2D)
3) …how often you are supposed to take this medication?	“It’s very easy. It’s once a week and I could take it anytime.” (F, 54y, T2D)
4) …the flexibility of taking this medication?	“That means how satisfied I am with the flexibility of taking this medication…I have the pump, I don’t have to stop to inject myself, look for privacy, or have to go into a bathroom, so this is more flexible, because I just got to hold it in my hand, and it’s giving me an insulin shot, wherever I’m at, so it’s very flexible having a pump and taking this.” (F, 67y, T1D) “It’s asking me basically, how am I liking taking the medication on my terms, my time. I really like it, taking it on my terms, my time. It’s extremely satisfying that I don’t have to take it at a certain time of the day, night, or whatever it be. If I forget to take it in the morning, I can do it in the day or I can do it in the evening, whichever, if I forgot or if I’m busy.” (F, 54y, T2D)
5) …the convenience of this medication?	“The injection is just, you know, very convenient, very easy to take anywhere, any time.” (M, 53y, T2D) “If it fits into my schedule.” (F, 74y, T2D) “Convenience of the medication itself. The convenience is you don’t have to pick it up every week from the pharmacy. You get the month’s supply. The convenience of doing it yourself, versus going to the doctor or the pharmacy to give you the injection. You’re able to do this in your own comfort of your home. To me, that’s a plus. That’s extremely satisfying. You’re not having to depend on someone else. It’s there to your disposal to use.” (F, 54y, T2D)
6) …how well you tolerate this medication (for example, if you experience side effects)?	“Am I having any side effect is what it meant to me. Extremely satisfied, because I’ve had none. No side effects.” (M, 53y, T2D) “It’s asking me do I physically tolerate the medication? How well do I tolerate it, with having any kind of illness or sickness or side effects. So it’s asking how does my body respond to the medication. How do I tolerate the medication, and do I have side effects.” (F, 59y, T2D)^c^
7) …the effect of this medication on your blood glucose control?	“The effect of your medication on your blood glucose control. My A1Cs and everything are very good, and it’s under control. So I’m extremely satisfied.” (M, 53y, T2D)
8) …the effect of this medication on aspects of your health **other than blood glucose**?	“The effect of this medication on aspects of your health other than blood glucose? It’s actually getting me to the point where I can get off some of my other medications, because I’ve lost a lot of weight. So I’ve been extremely satisfied.” (M, 53y, T2D)
9) …the effect of this medication on your weight?	“Do you feel that this medication has made you gain or lose weight, and if so, how do you feel about it?… I put neither on this one as well, just because I have had no effect.” (F, 25y, T1D) “Did it have any effects on my weight? Did I decrease, increase? I think one of the effects the medication was supposed to have was it was supposed to make, help me lose weight. I did drop a couple of pounds, but I don’t think that I dropped a lot. So I think I gave it a very dissatisfied or maybe a somewhat dissatisfied with that.” (F, 46y, T2D) “This question means, are you satisfied with the effect of this medication on your weight? It doesn’t mention positive or negative effects, just what is the effect of the medication on your weight. I answered extremely satisfied because I have been able to lose weight while being on this medication. So I have had a positive effect from this medication on my weight.” (F, 59y, T2D)^c^
10) …the effect of this medication on the time you spend thinking about food?	“I utilize the pump. Before, when I took shots, I probably thought about food more, because what I was going to eat, I had to do calculations and things like that, whereas with the pump, it’s so much more automatic. I do think about, there’s a difference sitting down having a bowl of pasta than there is sitting down having cottage cheese and fruit because some carbohydrates… I really don’t think about foods so much as I did before when I had to do manual injections… I don’t have to think about it so much anymore, so I’m very satisfied with that aspect.” (F, 66y, T1D) “To me, it means, do you have food on your mind all the time? Are you craving certain things? How much time do you spend thinking about what you’re going to eat?… Extremely satisfied because I don’t think about food. I don’t have cravings. I can eat my meals. I can choose healthier meals because I’m not craving something that would be more detrimental to my diet, with having diabetes.” (F, 59y, T2D)^c^
11) …the effect of this medication on your eating habits?	“I put very satisfied because I think it has helped with my eating habits… I eat healthy and less than I used to, before I had diabetes.” (M, 20y, T1D) “It’s asking your eating habits. Do you snack a lot? Do you always want to eat in between meals? Can you wait from breakfast to lunch or do you have to have a snack? Your eating habits, do you eat your three meals a day or not. So I feel like it’s asking is your appetite suppressed or are you able to eat your normal meals without having snacks all day long or snacking all day.” (F, 59y, T2D)^c^
12) …the effect of this medication on your energy level?	“It’s asking has this medication either negatively affected your energy level or positively affected your energy level? …I chose extremely satisfied … because of weight loss and your blood sugar staying normal and within limits, you don’t have the tiredness associated with the issues of having, being associated with high blood sugar and extreme weight.” (F, 59y, T2D)^c^
13) …the effect of this medication on your daily activities?	“Just normal daily activities…Getting up, showering, going through, enjoying whatever you need to get done in the day, housework, work, whatever.” (F, 59y, T2D)^c^ “Does this medication affect your daily activities, either positive or negative. If you’re sick from taking a medication, then that would impact you negatively. Like maybe you would have to call in sick from work, or you’re not able to function the way you normally would. So that’s what I feel like it’s asking, how does it affect your daily activities? Mine are I go to work every day, I go home every day. So I haven’t had any impact because I haven’t had any negative effects, like illness or anything like that.” (F, 59y, T2D)^c^
14) …the effect of this medication on your social activities and relationships?	“Going out with my family, going to social events, interacting with my spouse, dinner plans, social plans. That’s what I look at it as….If I felt fatigued and just the way the medication was making me feel, I just didn’t want to do any of those things…That’s what the medication did to me at the very beginning. I just had no motivation. Not wanting to do, not wanting to eat. Just kind of push myself through the day. But now, I’m somewhat satisfied getting there to be very satisfied.” (F, 54y, T2D) “Social activities and relationships obviously would mean around friends, around family, and around your significant other, if you have one. That’s what it means to me, the effect, is there any effect coexisting with these people? Does the medication interfere positively or negatively around things like that?… I put neither dissatisfied nor satisfied. Meaning that for me, personally, I don’t see any problems. It’s at a zero for me. It’s not satisfying and it’s not dissatisfied because it doesn’t really affect my social activities, whether negatively or positively.” (M, 60y, T2D)
15) Overall, how satisfied are you with this medication?	“Overall, it’s asking how do I feel taking the medication, the device of the medication, the doses of the medication, the side effects of the medication. That’s what it rounds out for me. For me, I put ‘very satisfied.’” (F, 54y, T2D)

Abbreviations: F = female; M = male; PSMD = Patient Satisfaction with Medication for Diabetes; T1D = type 1 diabetes; T2D = type 2 diabetes; y = years

^a^Items 1 to 14 have the same item stem: “In the past 7 days, how satisfied have you been with…”

^b^The item interpretations were provided in response to questions from interviewers such as “In your own words, what does this question mean to you?” and “What is this question asking?”

^c^The sample included two participants with the same combinations of age (59), gender (female), and type of diabetes (T2D)

Two participants had difficulty understanding the item on satisfaction with medication frequency as it appeared in the first two rounds of cognitive interviews (*How often you are supposed to take this medication*). The participants did not connect this item to the stem (*In the past 7 days, how satisfied have you been with…*) and therefore thought the item should be answered with a numerical response rather than a rating of satisfaction. To address this confusion, an ellipsis was added to the beginning of each item on the questionnaire in the third round of interviews to emphasize the connection to the item stem mentioning satisfaction. After this revision, all remaining participants were able to answer this item as intended without difficulty.

The item assessing satisfaction with treatment side effects required multiple revisions. This item was intended to evaluate the respondent’s satisfaction with treatment side effects regardless of whether side effects were experienced. The item initially asked participants how satisfied they had been with “the side effects of this medication,” but this was misinterpreted by a participant who thought it was asking about satisfaction with the general side effect profile of the medication rather than her personal experience with side effects. The item was revised to ask about satisfaction with “how well you tolerate this medication,” but two participants did not understand the term “tolerate” in this context. Therefore, the final version of the item included additional clarification: “…how well you tolerate this medication (for example, if you experience side effects)?” All participants completing this final version of the item understood it as intended.

Two items assessing satisfaction with the effect of medication on overall health and diabetes management were removed from the draft PSMD during the cognitive interviews. Participants reported that these items were redundant with other items on the questionnaire and difficult to answer.

Results of the cognitive interviews suggest that the final instrument is clear, comprehensible, and relevant to patients. The final PSMD items emerging from this phase of instrument development are presented in Fig. 2.

### PSMD emerging from this study

The version of the PSMD following refinements made based on the cognitive interviews includes 14 items assessing satisfaction with specific medication attributes and one global item assessing overall treatment satisfaction. Cognitive interview results suggest that all items apply to both T1D and T2D. Each item has seven response options, and the recall period is the “past 7 days.” The items are presented in Fig. 2 and Table 3.

The PSMD will be available for free upon contacting copyright@lilly.com. It is intended for broad use in clinical trials, academic research, and clinical practice, and it will be made available with permission.

## Discussion

In concept elicitation interviews, patients with T1D and T2D reported aspects of treatment that contribute to their satisfaction or dissatisfaction with medication for diabetes. Results of these qualitative interviews were considered along with relevant literature and clinician input when drafting a new PRO measure to assess patient satisfaction with diabetes medication. This measure, called the PSMD, was refined based on cognitive interviews with patients with T1D and T2D, and results suggest that the final instrument is clear, comprehensible, and relevant to patients. Overall, this qualitative research supports the content validity of the PSMD.

While previous PRO measures of treatment satisfaction are available for use in patients with diabetes, the PSMD is the first to be developed based on input from a sample that includes patients receiving the widely prescribed GLP-1 receptor agonists, as well as other common treatments like insulin and oral agents (Table 1). The new PRO measure emerging from this qualitative research includes a global item assessing overall treatment satisfaction and 14 items evaluating satisfaction with attributes of treatment for diabetes, such as blood glucose control, side effects, treatment process, and the impact of treatment on daily activities, social activities, and relationships. In addition, it includes concepts that are specifically relevant to newer medications for diabetes, including GLP-1 receptor agonists and a dual GIP/GLP-1 receptor agonist. These concepts, which were reported by patients in this study, include impact on weight, energy, and appetite/eating behavior. The addition of these concepts may allow for more sensitive assessment of diabetes treatment satisfaction in future clinical research. Based on input from the study participants, the PSMD appears to be relevant for patients receiving a broad range of prescribed medications, including oral medication, insulin, GLP-1 receptor agonists, and a dual GIP/GLP-1 receptor agonist.

The results should be considered in the context of the study’s limitations. Similar to all qualitative studies conducted for PRO instrument development, the current study was conducted with a relatively small sample size. While the current sample size is generally considered sufficient for a qualitative study of content validity [40], the 45 study participants may not be representative of the broader sample of patients with T1D and T2D. A sample of this size cannot provide insight into potential age, geographic, cultural, or clinical differences among patients or generalizability to the broader population. To mitigate this limitation and maximize generalizability, efforts were made to include patients receiving a variety of treatments to ensure that the new PRO measure would capture concepts important to a range of patients.

When developing items for the PSMD, there was a trade-off between specificity of the content and applicability to patients receiving a variety of treatments. Therefore, the items were designed to be specific enough to assess the treatment attributes that are important to these patients, yet general enough to apply to various medications for both T1D and T2D. Most items assess specific content and were interpreted similarly by all participants. For example, all respondents had a similar interpretation of the item assessing the effect of medication on blood glucose control. However, a few items were intentionally general to apply to all respondents, regardless of their specific treatment. For example, instead of including an item on dose adjustments, which is only relevant to patients treated with insulin or other adjustable medications, the PSMD includes an item assessing the more general concept of “flexibility.” This allows patients to interpret the item in a way that is relevant to their current treatment. These general items could be considered both a limitation (i.e., interpretation of the item may vary across patients) and a strength (i.e., broader applicability across patients) of the PSMD. Future research can evaluate the performance of each item, and the PSMD may be refined depending on psychometric results.

It should also be acknowledged that all three clinicians whose recommendations were considered when developing the patient interview guide were endocrinologists. Other members of the diabetes care team, as described in recommendations from the American Diabetes Association, were not included in this step. For example, important healthcare professionals may include diabetes education specialists, primary care clinicians, nurses, dietitian nutritionists, exercise specialists, and pharmacists [62]. It is possible that other types of healthcare professionals may have identified additional concepts not suggested by the three clinicians.

The current qualitative study is the first step in the development of the PSMD, and further research is needed to refine and assess this new questionnaire. Psychometric analysis of data from a larger sample can focus on item performance, possible item reduction, subscale identification, and a scoring algorithm, as well as the instrument’s reliability and validity. Additionally, longitudinal data, such as baseline and endpoint data from a clinical trial, may be used to examine the PSMD’s responsiveness to change and identify the minimum score difference that is considered important to patients.

## Conclusions

As new medications for diabetes are developed and tested, it is important to consider the extent to which patients are satisfied with these treatments. Ideally, treatment satisfaction would be assessed with a PRO measure designed to be relevant to patients receiving newer classes of treatment. In this study, the PSMD was developed based on the perceptions of patients treated with a broad range of medications for diabetes, including newer medications like GLP-1 receptor agonists and a GIP/GLP-1 receptor agonist. Following psychometric validation in larger samples, this new questionnaire may be useful for assessing patient satisfaction with medication in clinical trials. The PSMD may also be administered in clinical settings to efficiently assess a patient’s experience with their current medication.

## Data Availability

Study data and materials are available upon reasonable request. The PSMD will be available for free upon contacting copyright@lilly.com.

## Abbreviations

FDA: Food and Drug Administration
GIP: Glucose-dependent insulinotropic polypeptide
GLP-1: Glucagon-like peptide-1
HbA1c: Glycated hemoglobin
PSMD: Patient Satisfaction with Medication for Diabetes
PRO: Patient-reported outcome
SATMED-Q: Treatment Satisfaction with Medicines Questionnaire
TSQM: Treatment Satisfaction Questionnaire for Medication
T1D: Type 1 diabetes
T2D: Type 2 diabetes
